# Segmental Bone Reconstruction by Octacalcium Phosphate Collagen Composites with Teriparatide

**DOI:** 10.1089/ten.tea.2020.0150

**Published:** 2021-05-13

**Authors:** Keiko Matsui, Tadashi Kawai, Yushi Ezoe, Toshiki Yanagisawa, Tetsu Takahashi, Shinji Kamakura

**Affiliations:** ^1^ Division of Oral and Maxillofacial Surgery, Tohoku University Graduate School of Dentistry, Sendai, Japan.; ^2^ Division of Oral and Maxillofacial Surgery, School of Dentistry, Iwate Medical University, Morioka, Japan.; ^3^ Bone Regenerative Engineering Laboratory, Graduate School of Biomedical Engineering, Tohoku University, Sendai, Japan.

**Keywords:** octacalcium phosphate, collagen, teriparatide, bone regeneration, translational medical research

## Abstract

Octacalcium phosphate and collagen composite (OCPcol) demonstrated superior bone regeneration and has been commercialized recently in Japan. Teriparatide (TPTD) is a bioactive recombinant form of parathyroid hormone that is approved for osteoporosis treatment. Because mandibular bone reconstruction after segmental resection is a key clinical problem, it was examined whether single-dose local administration of OCPcol with TPTD can affect recovery after this procedure. OCPcol was prepared, and a commercially available hydroxyapatite and collagen composite (HAPcol) was used as a control. A 15 mm length segmental bone defect was made in the mandibular region of male beagle dogs. The experimental animals were divided in four groups. OCPcol treated with TPTD (OCPcol + TPTD), OCPcol, HAPcol treated with TPTD (HAPcol + TPTD), or HAPcol was implanted into the defect. The radiopaque areas of the implanted site were measured and statistically analyzed, and histological examination was performed after 6 months. The value of radiopaque area in total region of OCPcol + TPTD was highest (90.8 ± 7.3 mm^2^), followed in order by OCPcol (49.3 ± 21.8 mm^2^), HAPcol + TPTD (10.6 ± 2.3 mm^2^), and HAPcol (6.4 ± 2.3 mm^2^), and that of OCPcol + TPTD was significantly higher than that of HAPcol + TPTD or HAPcol. All segmented mandibles of OCPcol + TPTD and a part of those of OCPcol were bridged with newly formed bone, whereas no bone bridges were observed in HAPcol + TPTD or HAPcol. These results suggested that OCPcol treated with TPTD enabled bone reconstruction after segmental mandibular resection more than other three groups.

**Impact statement:**

Octacalcium phosphate and collagen composite (OCPcol) is a bone regenerative material that has been recently commercialized in Japan. Teriparatide (TPTD) is a recombinant form of parathyroid hormone, and is used for the treatment of osteoporosis. It was investigated whether single-dose local administration of OCPcol with TPTD can repair a canine segmental bone defect, which was supposed to be an intractable bone defect. Compared with OCPcol, hydroxyapatite, and collagen composite (HAPcol) with TPTD, or HAPcol, because OCPcol with TPTD quickly enhanced bone regeneration and bridged the defect, it would be a candidate for the repair of intractable bone defect.

## Introduction

In the field of reconstructive surgery, the reconstruction of refractory bone defects, such as segmental bone defects that follow the resection of tumors, is a key problem.^1,2^ To reconstruct large bone defects that cause functional disturbances, autologous bone grafting has been adopted as a gold standard, where bone material is collected from the patient and transplanted to the treatment site or as free flaps with bone.^3^ However, these procedures impose a large burden on patients, because they involve a second surgery, only a limited amount of bone can be collected, and such surgery requires hospitalization for treatment.^4,5^

Therefore, the development of biomaterials as bone substitutes would overcome the disadvantages of autologous bone grafting.^6^ In fact, hydroxyapatite [HAP; Ca_10_(PO_4_)_6_(OH)_2_] and β-tricalcium phosphate [β-TCP; Ca_3_(PO_4_)_2_] have been used in clinical situations for many years, and these have been further developed and used clinically with new composite materials, such as hydroxyapatite and collagen composite (HAPcol).^7–9^ However, these materials cannot achieve reproducible bone regeneration in refractory bone defects, and autologous bone grafting is still the standard treatment for such defects.^10^

Octacalcium phosphate [OCP; Ca_8_H_2_(PO_4_)_6_ · 5H_2_O] is classified among the calcium phosphates, similar to HAP or β-TCP, and it exhibits excellent bone regenerative properties and bioresorbability.^11^ Although OCP is a synthetic bioresorbable material similar to β-TCP, OCP also has unique features. In animal studies, it stimulated osteogenesis by osteoblastic cells and/or committed osteoprogenitors,^12^ and it can serve as a core for initiating bone regeneration if implanted in a bone defect.^13^ Unfortunately, it has been difficult to advance the clinical application of OCP because of its poor moldability and handling performance.^14^

Consequently, OCP and collagen composite (OCPcol) was developed to overcome these limitations.^14^ Sponge-like OCPcol disks were prepared using granules of OCP and atelocollagen derived from porcine dermis. It has promoted osteogenic differentiation and angiogenesis,^15^ and exhibited excellent bone regenerative properties in preclinical studies.^16–18^ Also, it showed good safety and efficacy in clinical situations,^19–22^ and was recently commercialized in Japan.^23,24^

Parathyroid hormone (PTH) regulates the metabolism and functions of calcium and phosphate,^25^ and teriparatide (TPTD) is a recombinant form of PTH consisting of the bioactive portion of the N-terminal fragment comprising 34 amino acids.^26^ TPTD is an authorized anabolic drug for the treatment of osteoporosis,^27^ and its intermittent administration is used to increase trabecular bone.^28,29^ Recently, it was reported that OCPcol with a local single administration of TPTD (1 or 0.1 μg), which is a relatively low dose similar to that for the treatment for osteoporosis, enhanced bone repair of a critical-sized bone defect.^30,31^ Hence, the present study investigated whether single-dose local administration of TPTD with OCPcol achieved bone reconstruction if implanted into a canine segmental mandibular bone defect.

## Materials and Methods

### Preparation of OCPcol and HAPcol

The preparation of OCPcol was described previously.^30^ In brief, OCP was prepared by direct precipitation, and sieved granules (particle sizes 300–500 μm) of OCP were produced. Collagen was prepared from NMP collagen PS (Nippon Meat Packers, Tsukuba, Ibaraki, Japan), a lyophilized powder of pepsin-digested atelocollagen isolated from porcine dermis. OCP granules were mixed with concentrated collagen, with 77% of the weight percentage as OCP in OCPcol. The OCPcol mixture was then lyophilized, and a disk was molded (diameter 9 mm, thickness 1.5 mm). OCPcol disks were prepared using a dehydrothermal treatment (150°C, 24 h) in a vacuum drying oven. OCPcol disks were sterilized using electron beam irradiation and provided from TOYOBO Co. Ltd. (Osaka, Japan).

Commercially available bone substitutes of HAPcol (Refit^®^, PC-1112, φ11 × 12 mm; Hoya Technosurgical Corp., Tokyo, Japan) were purchased. It was composed of 80% by weight of low-crystalline HAP and atelocollagen derived from porcine dermis, and is reported to be absorbed during the process of bone remodeling, although the conventionally used HAP was hardly resorbed in the vital tissue.^32^

### Preparation of TPTD solution

**FIG. 1.**
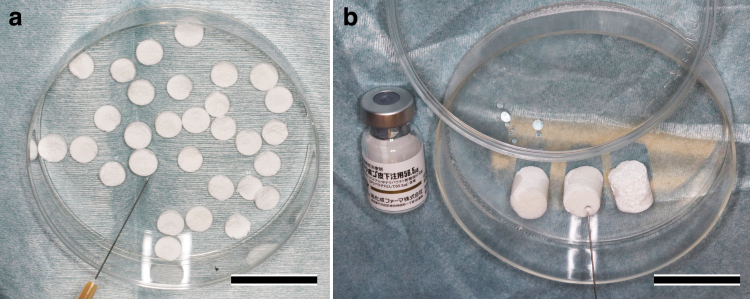
Preparation of OCPcol + TPTD and HAPcol + TPTD. Lyophilized teriparatide acetate (56.5 μg), reconstituted with 1.0 mL of saline, was applied immediately before implantation of OCPcol **(a)** or HAPcol **(b)**. Bars: 30 mm. OCPcol, octacalcium phosphate and collagen composite; HAPcol, hydroxyapatite and collagen composite; TPTD, teriparatide. Color images are available online.

Chemically synthesized teriparatide acetate, the active ingredient of TERIBONE^TM^ Inj. 56.5 μg (Asahi Kasei Pharma Corp., Tokyo, Japan), was used. Lyophilized teriparatide acetate was reconstituted with 1.0 mL of saline at a concentration of 56.5 μg/mL immediately before implantation with OCPcol or HAPcol (Fig. 1).

### Experimental animals

Twenty male beagle dogs (age: 18 months; Kitayama Labs Co., Ltd., Ina, Japan) were used for the experiments. The principles of laboratory animal care, as well as national laws, were followed. The Institutional Animal Care and Use Committee of the Tohoku University Environmental and Safety Committee approved all procedures in this study, under approvals 2015-Dentistry Animal-008 and 2018-Dentistry Animal-001 (2018DnA-001).

In the experiment facilities of Tohoku University, the temperature and humidity were managed. The laboratory animals were bred using a cage in the experiment facilities. The laboratory animals were managed by a contracted veterinarian, and the laboratory animals engaged in periodic activity and received examination regularly. After surgery, the animals received a soft diet and had free access to drinking water. We carefully checked for the presence of harmful phenomena, such as wound compartment infection, decreased appetite, and death.

### Implantation procedures

Surgical procedures were divided into two stages. The first stage of surgery was the extraction of mandibular teeth, and the second stage of surgery was the creation of a critical-sized segmental mandibular bone defect with implantation of bone substitutes with or without TPTD. Both procedures were performed under general anesthesia, which was administered using intravenous sodium pentobarbital (25 mg/kg), followed by intramuscular atropine sulfate (0.5 mg) and ketamine hydrochloride (20 mg/kg) with 50 mg intramuscular injection of analgesics (ketoprofen, Carvisken^®^; Kissei Pharmaceutical Co. Ltd., Matsumoto, Japan). Local anesthetic (2% lidocaine with 1/80,000 epinephrine) was injected after disinfection of the operating field.

The first stage of surgery was the extraction of four premolars (P1–P4) from the left mandible using elevator or tooth extraction forceps. This procedure may lead to a reduction in intraoral infection due to damage to the oral mucosa during the second stage of surgery. Before the second surgery, a 3- to 4-month interval was included to allow healing of the extracted alveolus.

In the second stage of surgery, five experimental animals per group were randomly divided into four groups: OCPcol + TPTD, OCPcol, HAPcol + TPTD, and HAPcol. Each group was implanted with specimens as follows; the OCPcol + TPTD or OCPcol group was implanted with 30–43 disks of OCPcol (9 mm φ × 1.5 mm), and the HAPcol + TPTD or HAPcol group was implanted with three pieces of HAPcol (11 mm φ × 12 mm). The implanted volume of OCPcol was equivalent to that of HAPcol. In addition, the OCPcol + TPTD or HAPcol + TPTD group received 56.5 μg/mL of reconstituted TPTD.

**FIG. 2.**
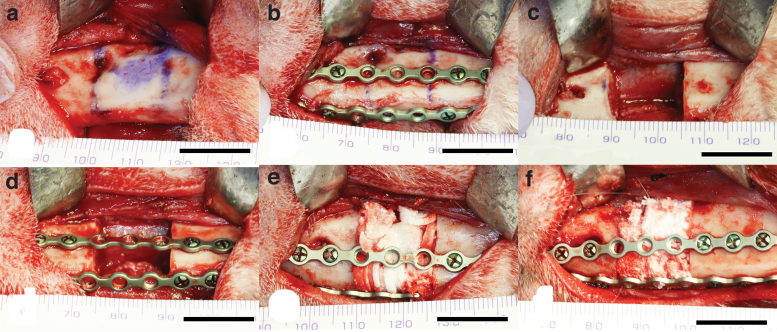
Second stage surgery of segmental mandibular resection. First, a 15 mm resection area was marked **(a)**. Then, two titanium mini plates were tied-in and drilled to secure the continuity of mandible after surgery **(b)**. Next, a critical-sized segmental mandibular bone defect was made under continuous saline buffer irrigation **(c)**. After the segmented mandible was repositioned by fixing the titanium mini plates **(d)**, the test materials [OCPcol + TPTD, OCPcol **(e)**, HAPcol + TPTD, or HAPcol **(f)**] were implanted into the defect. Bars: 15 mm. Color images are available online.

A 15 mm length segmental bone defect was made in the left mandibular premolar region. After additional local anesthesia, a skin incision in the left submandibular region (about 5 cm length) was made. Then, a periosteal incision was made in the lower border of the mandible, and the periosteum was circumferentially ablated through the premolar region. Then, a 15 mm length of the resected area was assumed and marked (Fig. 2a). Then, two titanium mini plates were tied-in and drilled on the buccal side and the side of the mandibular lower edge to secure the continuity of the mandible after segmental surgery (Fig. 2b).

After this, critical-sized segmental mandibular bone defects were made by using a Lindemann drill with a micromotor system (Elan EC, AESCULAP^®^, Tuttlingen, Germany) under continuous saline buffer irrigation (Fig. 2c). Thereafter, the segmented mandible was repositioned by fixing the titanium mini plates adjusted (Mandibular fixation plates 16 holes; Stryker Leibinger GmbH and Co. KG, Freiburg, Germany) with bone screw (2.0 Mandibular Bone Screws, Cross Pin, 2.0 × 8 mm, or 2.0 × 10 mm; Stryker Leibinger GmbH and Co. KG) (Fig. 2d), the specimens (OCPcol + TPTD, OCPcol, HAPcol + TPTD, and HAPcol) were implanted into the defect (Fig. 2e, f), and the ablated periosteum and skin were repositioned and sutured.

In the present study, the doses of TPTD were 4.61 ± 0.27 μg/kg in the OCPcol + TPTD group and 4.33 ± 0.13 μg/kg in the HAPcol + TPTD group when converted to an average weight in each group at the second surgery. To prevent infection or swelling, 1000 mg of flomoxef sodium (Flumarin^®^; Shionogi and Co. Ltd., Osaka, Japan) was used by intravenous drip during the operation, and 125 mg of methylprednisolone sodium succinate (Decacort^®^; Sawai Pharmaceutical Co. Ltd., Osaka, Japan) was used by intravenous injection during the operation. In addition, 100 mg of cefcapene pivoxil hydrochloride hydrate (Flomox^®^; Shionogi and Co., Osaka, Japan) was used by oral administration for 3 days after surgery as well as first stage surgery.

### Intraoral radiography and oral examinations

Intraoral radiography and oral examinations were performed immediately and every month after implantation under anesthesia with sodium pentobarbital (25 mg/kg). Intramuscular atropine sulfate (0.25 mg) and ketamine hydrochloride (20 mg/kg) were used to restrict the excessive movements of animals during intraoral radiography and oral examinations. Dental radiographs in lateral and axial views were taken using dental radiography (PORT-Xfil; J. MORITA Corp., Suita, Osaka, Japan) with instant film for occlusal radiography (Hanshin Technical Laboratory, Ltd., Nishinomiya, Hyogo, Japan) under standardized conditions (60 kV, 2 mA, 0.8 s. [lateral view] or 2.0 s [axial view]).

At 6 months after implantation, the experimental animals were euthanized by intravenous injection of an overdose of sodium pentobarbital after intramuscular injection of ketamine hydrochloride.

### Radiographic examinations

After sacrifice, the mandible and surrounding tissues were resected and fixed in 10% formalin neutral buffer solution, pH 7.4. The excised mandibles were radiographed by a microradiography unit (Softex M-60; Softex Co., Ltd., Ebina, Kanagawa, Japan) with X-ray film (FR; Fuji Photo Film, Tokyo, Japan) in standardized conditions (45 kV, 1.5 mA, 2 min).

### Microcomputed tomography examination and radiomorphometric analysis (Fig. 3)

The specimens were scanned using an *in vivo* microcomputed tomography (micro-CT) system (Latheta LCT-200; Hitachi Aloka Medical, Tokyo, Japan). The treated defects were scanned in 240 μm thickness for slices and 120 μm pixel size. The images of micro-CT were acquired in standardized conditions (50 kVp, 500 μA, 3.6 ms). The CT images were classified into 256 gray scale, and only the whitest areas were extracted and their areas excluding titanium mini plates (mm^2^) were calculated.

**FIG. 3.**
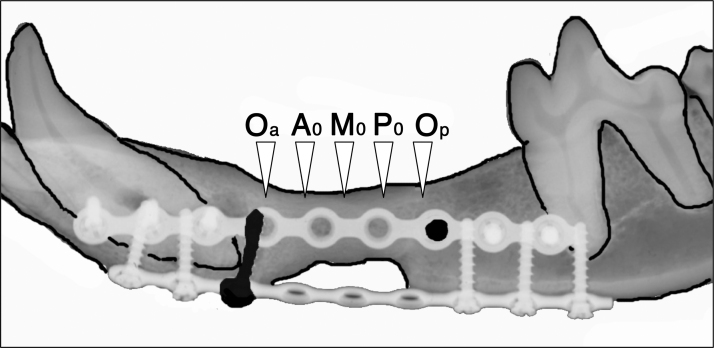
Radiomorphometric analysis of radiopaque figures from micro-CT image. By using micro-CT image, it was quantified the areas of radiopaque figures excluding titanium mini plates. The image was selected as follows: (1) the image of original anterior (Oa) was designated as the first image which disappeared the most posterior bone screw on the part of distal resected stump of anterior segment of original mandible. (2) The image of original posterior (Op) was designated as the just before image which appeared the most anterior bone screw on the part of medial resected stump of posterior segment of original mandible. (3) The center image between Oa and Op was designated as M_0_, and the two images before and behind of M_0_ was selected. These three figures were named Middle (M). (4) Likewise, the center image between Oa and M_0_ was designated as A_0_, and the two images before and behind of A_0_ was selected. These three figures were named Anterior (A). (5) The center image between Op and M_0_ was designated as P_0_, and the two images before and behind of P_0_ was selected. These three figures were named Posterior (P). Then, the radiopaque area excluding titanium mini plates on Anterior (A), Middle (M), Posterior (P), and Total (T), which was sum of A, M, and P, was quantified. micro-CT, microcomputed tomography.

The image was selected as follows: (1) the image of original anterior (Oa) was designated as the first image, which disappeared the most posterior bone screw on the part of distal resected stump of anterior segment of original mandible (Fig. 3). (2) The image of original posterior (Op) was designated as the just before image which appeared the most anterior bone screw on the part of medial resected stump of posterior segment of original mandible. (3) The center image between Oa and Op was designated as M_0_, and the two images before and behind of M_0_ were selected. These three figures were named Middle (M). (4) Likewise, the center image between Oa and M_0_ was designated as A_0_, and the two images before and behind of A_0_ were selected. These three figures were named Anterior (A). (5) The center image between Op and M_0_ was designated as P_0_, and the two images before and behind of P_0_ were selected. These three figures were named Posterior (P) (Fig. 3).

Then, the radiopaque area excluding titanium mini plates on Anterior (A), Middle (M), Posterior (P), and Total (T), which was sum of A, M, and P, was quantified by using ImageJ version 10.2 (National Institutes of Health, Bethesda, MD) public domain software.

### Statistical analysis

The radiopaque area was analyzed statistically using IBM SPSS statistics version 25 (IBM Corporation, Armonk, NY). All values are reported as mean ± standard error. The Kolmogorov–Smirnov test was used to investigate normal distribution of each group, and Levene's test was used to examine the homogeneity of variance between samples. Welch's analysis of variance was used to compare mean values of each group. Statistical significance was determined at *p* < 0.05. In cases where significant differences were detected between the mean values, Dunnett T3 *post hoc* test was performed.

### Histological examination

The undecalcified specimens were then fixed in 70% ethanol, stained with Villanueva bone stain, dehydrated in graded ethanol, and embedded in methyl methacrylate. Then, the central part of the created defect was sectioned coronally using a low-speed saw machine (Isomet 5000; Buehler, Lake Bluff, IL) with a diamond wafering blade. The sectioned wafers were mounted on plastic slides and were ground and polished until they were 20–30 μm thick and observed with a stereomicroscope (Leica S9D; Leica Microsystems Japan, Tokyo, Japan) with light board and a photomicroscope (Leica DM2500; Leica Microsystems Japan).

## Results

### Macroscopic view (Fig. 4)

In most cases (19/20: 95.0%), the body weights of experimental animals increased between implant surgery and 6 months after implantation. Although postsurgical weight loss was observed in one case in the OCPcol group, they were generally well and no eating disorders and postoperative infections were observed.

At 6 months after the second stage surgery, the alveolar shape of the OCPcol + TPTD implanted area had augmented with sufficient height and width in all cases. In contrast, the OCPcol group was polarized: one group indicated augmented alveolus with height and width maintained, and the other group demonstrated a narrow alveolar ridge that was concave toward the central part of the implanted area. However, there was an observed concave shape of the central part of the implanted area from the early postoperative period in the HAPcol + TPTD and HAPcol groups, and narrow alveolar ridges were sustained up to the end of the experiment.

Several postoperative complications were observed: postoperative infection associated with intraoral dehiscence or submandibular fistula was recognized in one of five (OCPcol + TPTD), two of five (OCPcol), three of five (HAPcol + TPTD), and two of five (HAPcol). In these cases, intraoral exposure of the titanium plate was observed in 0 of 5 (OCPcol + TPTD), 1 of 5 (OCPcol), 2 of 5 (HAPcol + TPTD), and 1 of 5 (HAPcol). In addition, titanium plate fracture was detected in 0 of 5 (OCPcol + TPTD), 1 of 5 (OCPcol), 3 of 5 (HAPcol + TPTD), and 1 of 5 (HAPcol).

### Radiographic examination (Figs. 5 and 6)

Immediately after operation, implanted disks formed from OCPcol or HAPcol showed hardly any radiopacity in the defect. At 6 months after implantation, a “bone bridge” was observed, which meant the closure of the resected bone defect with newly formed bone in all cases in the OCPcol + TPTD group (five of five) and 60% of cases in the OCPcol group (three of five). However, no bone bridges were observed in the HAPcol + TPTD and HAPcol groups (Fig. 5).

**FIG. 4.**
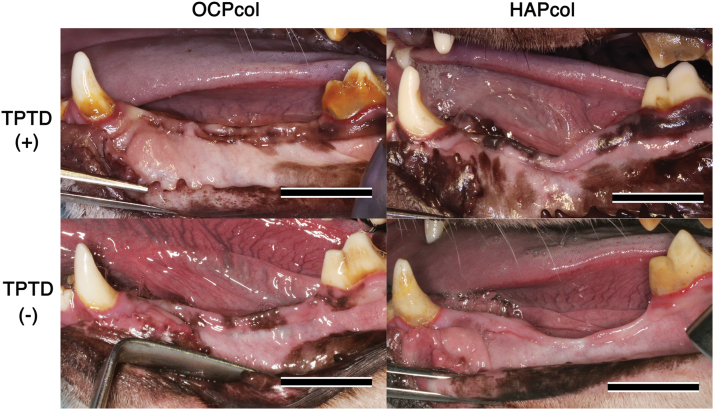
Macroscopic view at 6 months after second stage surgery. The alveolar shape of the OCPcol + TPTD implanted area had augmented with sufficient height and width in all cases. In contrast, the OCPcol group was polarized: one group indicated augmented alveolus maintaining their height and width, and the other group demonstrated an atrophic alveolar ridge. However, concavity of the central part of the implanted area was observed in all cases in the HAPcol + TPTD and HAPcol groups. Bars: 15 mm. Color images are available online.

**FIG. 5.**
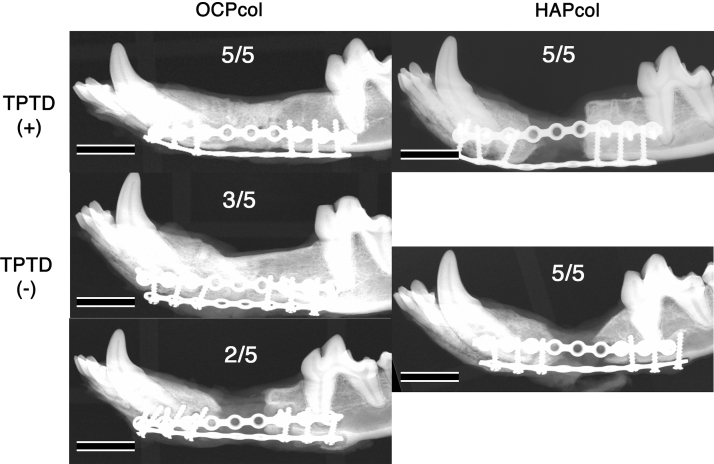
Radiographic lateral view at 6 months after second stage surgery. A bone bridge was observed, which meant the closure of the resected bone defect by newly formed bone in all cases in the OCPcol + TPTD group (five of five) and 60% of cases in the OCPcol group (three of five). However, no bone bridges were observed in the HAPcol + TPTD or HAPcol group. Bars: 15 mm.

**FIG. 6.**
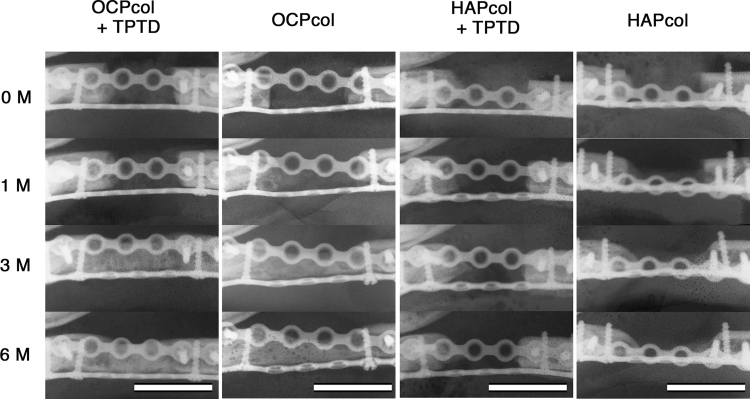
Sequential radiographic axial view after second stage surgery. In the OCPcol + TPTD group, well-defined radiolucency in the defect was observed immediately after operation, then the resected bone margin became indistinct at 1 month after operation. At 3 months after operation, there was less radiopacity throughout the defect, and the demarcation between newly formed bone and original bone was barely recognizable, and higher radiopacity was observed throughout the defect at 6 months after operation. In bone bridges formed after OCPcol implantation, radiopaque figure in the defect seemed to occur at a later stage than in cases with bone bridges in the OCPcol + TPTD group. In the HAPcol + TPTD or HAPcol groups, no radiopaque figure was observed in the central part of the bone defect, although a small amount of radiopacity increased from the resected bone margin as in cases without bone bridges in the OCPcol group. Bars: 15 mm.

In sequential radiographic examinations of bone bridges with OCPcol + TPTD group, well-defined radiolucency in the defect was observed immediately after operation, then the resected bone margin became indistinct at 1 month after operation. At 3 months after operation, there was less radiopacity throughout the defect, and the demarcation between newly formed bone and original bone was barely recognizable, and higher radiopacity was observed throughout the defect at 6 months after operation (Fig. 5). In the cases of bone bridges with OCPcol implantation, radiopaque regions in the defect seemed to occur at a later stage than in cases with bone bridges in the OCPcol + TPTD group (Fig. 6).

In contrast, in cases without bone bridges after OCPcol implantation, no radiopaque region originating from newly formed bone was observed in the central part of the bone defect throughout the observation period, although small amounts of radiopacity increased from the resected bone margin. Moreover, bone bridges were observed even in cases with postoperative inflammation such as redness or granulation in the OCPcol + TPTD and OCPcol groups. In the HAPcol + TPTD and HAPcol groups, no radiopaque figure was observed in the central part of the bone defect, although radiopacity increased slightly from the resected bone margin in cases without bone bridges after implantation (Fig. 6).

### micro-CT examination (Fig. 7)

**FIG. 7.**
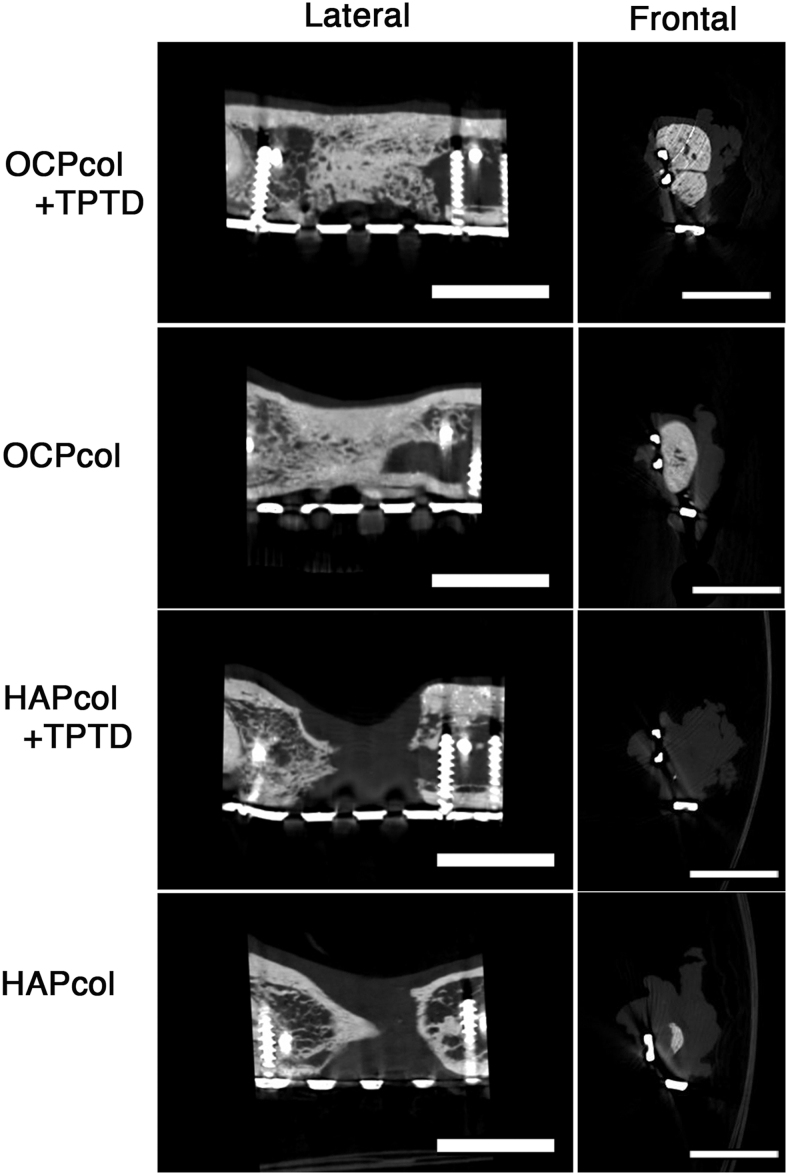
Lateral and frontal view of micro-CT at 6 months after second stage surgery. In cases with bone bridges in the OCPcol + TPTD or OCPcol group, lateral view indicated that the resected bone margin had sufficient amounts of radiopaque figure, and demarcation between the original bone and newly formed bone was indistinct. In cases implanted with HAPcol + TPTD or HAPcol, radiopaque figure originating from the margin of original bone was observed. In the cases with bone bridges in the OCPcol + TPTD or OCPcol group, frontal view indicated that radiopacity was uneven in the central part of the defect. Moreover, the outer periphery is dense and had higher radiopacity, whereas the inner portion had a trabecular-like structure and lower radiopacity. In cases in the HAPcol + TPTD or HAPcol group, radiopacity was barely recognized in the central part of the defect. Bars: 10 mm.

In cases with bone bridges in the OCPcol + TPTD and OCPcol groups, the segmented defects were occupied by sufficient amounts of radiopaque figures, and demarcation between the original bone and newly formed bone was indistinct in lateral sections (Fig. 7). Also, the radiopacity was uneven at the implant site in frontal sections. In addition, the outer periphery was dense and showed higher radiopacity, whereas the inner portion had a trabecular-like structure and lower radiopacity (Fig. 7). In cases without bone bridges in the OCPcol, HAPcol + TPTD, and HAPcol groups, radiopaque figure originating from the margin of the original bone was observed (Fig. 7). However, no bone bridges were achieved, and radiopacity in the central part of the defect was hardly recognized (Fig. 7).

### Radiomorphometric analysis (Figs. 8 and 9)

**FIG. 8.**
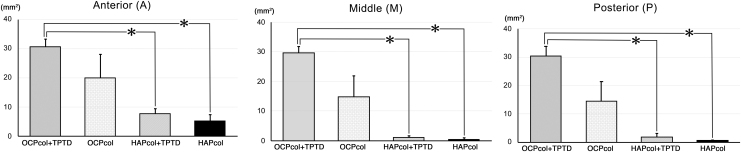
Radiopaque area in Anterior (A), Middle (M), and Posterior (P) region of resected mandible. The radiopaque area of OCPcol + TPTD was highest, followed in order by OCPcol, HAPcol + TPTD, and HAPcol in the three selected area (A, M, and P). Also, the radiopaque area of OCPcol + TPTD was significantly higher than that of HAPcol + TPTD or HAPcol in the three selected area (A, M, and P). *p < 0.05.

**FIG. 9.**
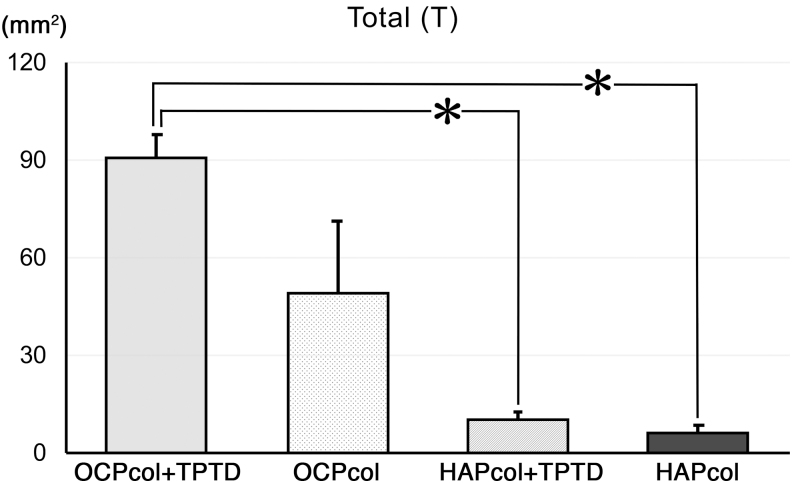
Radiopaque area in Total (T) region of resected mandible. The radiopaque area of OCPcol + TPTD was highest, followed in order by OCPcol, HAPcol + TPTD, and HAPcol. Also, the radiopaque area of OCPcol + TPTD was significantly higher than that of HAPcol + TPTD or HAPcol. *p < 0.05.

The radiopaque areas in Anterior (A) were 30.6 ± 2.5 mm^2^ (OCPcol + TPTD), 20.0 ± 8.1 mm^2^ (OCPcol), 7.8 ± 1.7 mm^2^ (HAPcol + TPTD), and 5.3 ± 2.0 mm^2^ (HAPcol), respectively. The radiopaque areas in Middle (M) were 29.7 ± 2.0 mm^2^ (OCPcol + TPTD), 14.8 ± 7.2 mm^2^ (OCPcol), 1.1 ± 0.62 mm^2^ (HAPcol + TPTD), and 0.48 ± 0.48 mm^2^ (HAPcol), respectively. The radiopaque areas in Posterior (P) were 30.4 ± 3.3 mm^2^ (OCPcol + TPTD), 14.5 ± 6.8 mm^2^ (OCPcol), 1.7 ± 1.3 mm^2^ (HAPcol + TPTD), and 0.60 ± 0.28 mm^2^ (HAPcol), respectively (Fig. 8). The radiopaque areas in Total (T) were 90.8 ± 7.3 mm^2^ (OCPcol + TPTD), 49.3 ± 21.8 mm^2^ (OCPcol), 10.6 ± 2.3 mm^2^ (HAPcol + TPTD), and 6.4 ± 2.3 mm^2^ (HAPcol), respectively (Fig. 9).

In A, M, P, and T, significant differences were detected between OCPcol + TPTD and HAPcol + TPTD and between OCPcol + TPTD and HAPcol (Figs. 8 and 9).

### Histological analysis (Figs. 10 and 11)

**FIG. 10.**
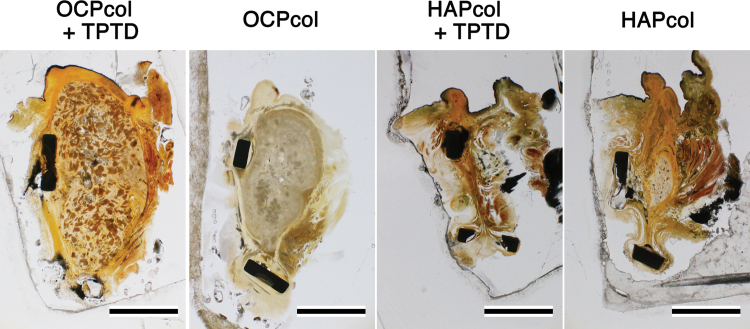
Histological overview of implants at 6 months. In OCPcol + TPTD or OCPcol group, with augmented alveolus, plentiful newly formed bone was amalgamated with the remaining implants in the central part of the created defect, although no or little bone was observed in HAPcol + TPTD or HAPcol group. Also, the amount of newly formed bone in OCPcol + TPTD group was more than that in OCPcol group. Villanueva bone stain, Bars: 5 mm. Color images are available online.

**FIG. 11.**
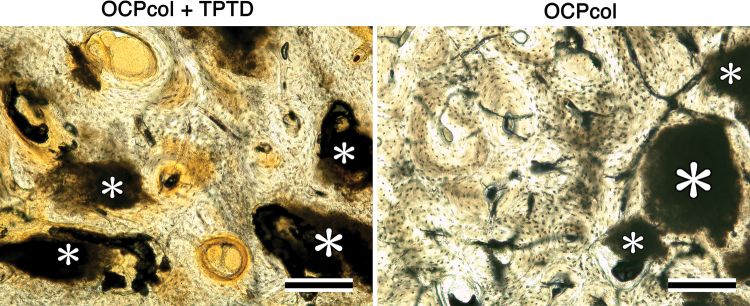
Histological examination of implants at 6 months. In OCPcol + TPTD or OCPcol group with augmented alveolus, the newly formed bone has a typical structure of osteon with osteocytes, and no demarcation was observed between the newly formed bone and the remaining implants (*). Villanueva bone stain, Bars: 200 μm. Color images are available online.

In OCPcol + TPTD or OCPcol groups with augmented alveolus, plentiful newly formed bone was amalgamated with the remaining implants in the central part of the created defect, although no or little bone was observed in HAPcol + TPTD or HAPcol group (Fig. 10). Also, the amount of newly formed bone in OCPcol + TPTD group was more than that in OCPcol group (Fig. 10). In OCPcol + TPTD or OCPcol group with augmented alveolus, the newly formed bone has a typical structure of osteon with osteocytes, and no demarcation was observed between the newly formed bone and the remaining implants (Fig. 11).

## Discussion

In the radiomorphometric analysis, the radiopaque area of OCPcol + TPTD was highest, followed in order by OCPcol, HAPcol + TPTD, and HAPcol in every selected area. Also, histological analysis in the central part of the created defect demonstrated that plentiful newly formed bone with the remaining implants was observed in OCPcol + TPTD or OCPcol group with augmented alveolus, whereas no or little bone was observed in HAPcol + TPTD or HAPcol group. The remaining implants were supposed to be irreversibly converted apatite from OCP,^33^ and it would be expected to replace by newly formed bone, because the implanted OCPcol was replaced by newly formed bone at 6 or 10 months after implantation.^34,35^

The present study demonstrated that all cases treated with OCPcol + TPTD achieved a bone bridge, which meant closure of the resected bone defect by newly formed bone, although 60% of cases treated with OCPcol achieved a bone bridge. However, the value of radiopaque area in OCPcol group was polarized, and no significant difference was detected between OCPcol and other three groups, including OCPcol + TPTD. These results suggest that OCPcol + TPTD group would exhibit excellent bone reconstruction after segmental mandibular resection than OCPcol group, if OCPcol was implanted with a single-dose local administration of TPTD.

In contrast, no bone bridges were observed in the HAPcol + TPTD and HAPcol groups, and significant differences of the radiopaque areas were detected between OCPcol + TPTD and HAPcol + TPTD and between OCPcol + TPTD and HAPcol in all selected areas. Although HAPcol used in this study had a different manufacturing process, it was reported that the bone regenerative properties of OCPcol were more prominent than those of HAPcol.^16^ In addition, it was confirmed that bone regeneration was enhanced if OCPcol was implanted with a single-dose local administration of TPTD.^30^ Consequently, it suggests that OCPcol + TPTD enhanced bone regeneration more than HAPcol + TPTD and HAPcol groups in this segmental defect, and a combination of OCPcol with TPTD may specifically lead to a synergistic effect on bone regeneration.

The alveolar shape of the implanted area was maintained in OCPcol + TPTD group and the cases with bone bridges in the OCPcol group, which had sufficient height and width during the experimental periods as well as immediately before the second stage surgery. In cases without bone bridges in the OCPcol, HAPcol, and HAPcol + TPTD groups, the concavity of the central part of the implant area was observed from the early postoperative period. No recovery of concavity was observed, and an atrophic narrow alveolar ridge was still present at the end of the observation period. It was suggested that macroscopic observation of alveolar shape would predict the prognosis in this experimental model similar to radiographic examination.

In sequential radiographic examinations in OCPcol + TPTD group and the cases with bone bridges in the OCPcol group, the implant site had well-defined radiolucency immediately after operation. The resected bone margin became indistinct after 1 month, and lower radiopacity was evident throughout the defect after 3 months. Then, the radiopacity increased at 6 months. It has reported previously that granules of OCP or OCPcol nucleated and enhanced bone formation,^13,14^ and implantation of OCPcol + TPTD or OCPcol was able to induce radiopacity throughout the defect at 3 months after operation, even though lower radiopacity was recorded.

In the radiomorphometric analysis, the values of radiopaque area of OCPcol + TPTD or OCPcol demonstrated as well as in the selected three areas (A, M, and P), although those of HAPcol + TPTD or HAPcol were tended to be lower than other area. This meant that bone regeneration with OCPcol + TPTD or OCPcol progressed simultaneously from the defect margin of the original bone and from inside of the implants, which was a representative feature of bone regeneration with OCPcol or OCPcol + TPTD. However, bone regeneration with HAPcol or HAPcol + TPTD might be mainly elicited from the defect margin of the original bone. In addition, the emergence of radiopacity in the OCPcol + TPTD group was faster than in the OCPcol group, because it was reported that OCPcol + TPTD enhanced bone regeneration more than OCPcol.^30^

The results of bone reconstruction in the OCPcol group were polarized as cases with or without bone bridges after OCPcol implantation. Because the speed of bone formation with OCPcol was slower than with OCPcol + TPTD, the resorption of OCPcol as a scaffold might reduce bone reconstruction after segmental mandibular resection, if the degradation of implanted materials was faster than that of bone formation. It was also suggested that the driving force of bone formation in the early postoperative period may be important for bone reconstruction of this canine segmental bone defect of the mandible.

In contrast, no bone bridge was observed in cases in the HAPcol or HAPcol + TPTD group. In these cases, it was observed that soft tissues at the implant site shrank by lowering or narrowing the alveolar ridge from the early postoperative period. It was supposed that the collapse of the implants as a scaffold may cause the failure of bone reconstruction in this model, when rapid degradation of the implant materials can considerably exceed bone formation. Therefore, it may be unclear whether bone regeneration was sufficiently initiated by adding TPTD in this situation. Consequently, OCPcol + TPTD specifically achieved better bone reconstruction in this experimental model than HAPcol + TPTD.

In cases with bone bridges after OCPcol + TPTD or OCPcol on micro-CT, it was difficult to recognize the demarcation between newly formed bone and original bone as well as the long-term prognosis of implanted OCPcol.^34,35^ It was demonstrated that the original bone was tightly united with the newly formed bone derived from OCPcol, and the repaired bone from the implantation of OCPcol might be difficult to refracture at the margin of the original bone rather than conventional bone substitutes.

In addition, uneven radiopacity was seen in the frontal sections of micro-CT of cases with bone bridges in the OCPcol + TPTD and OCPcol groups at 6 months after operation. These consisted of a dense outer periphery and less dense inner portion with a trabecular-like structure. Because these findings seemed to be similar to previous reports,^34,35^ and newly formed bone in the OCPcol + TPTD and OCPcol groups could be remodeled in a physiological manner.

Although the general conditions of the experimental animals were relatively stable, and no eating disorders were observed through the experiments, several postoperative complications were observed, including intraoral dehiscence, intraoral exposure of the titanium plate, or titanium plate fracture. The cause of postoperative infection may be assumed by the degradation and resorption of the implanted materials preceded by bone regeneration inside implants rather than the implanted materials causing infection that spread to surrounding tissues.

The degradation and resorption of the implants may increase the repetition of damage to the edges of preexisting bone or the oral mucosa covering implant during jaw movement, such as mastication. Consequently, these actions may initiate and spread postoperative infection by intraoral dehiscence. Although no bone bridge was attained if the reconstructed alveolar shape collapsed in the early postoperative period, bone bridges were still observed in cases associated with postoperative infection.

Titanium plate fracture may occur if excessive external force is applied.^36^ Although a reconstruction plate was used to fix the segmented mandible in preliminary experiments as well as clinical cases, the procedures prevented the establishment of an experimental method due to the occurrence of intraoral dehiscence and subsequent postoperative infection. Such infection may be caused by more intensive stimulus between the oral mucosa and the reconstruction plate, because the reconstruction plate was significantly thicker than the titanium mini plate used in this experiment. Therefore, titanium mini plates were adopted to fix the resected mandible in this study. Nevertheless, a more delicate experimental method should be established to eliminate the possibility of postoperative infection or plate fracture.

Although it was confirmed that segmental bone defects were repaired with newly formed bone if OCPcol was implanted with single-dose local administration of TPTD (4.61 ± 0.27 μg/kg) in this experimental model, further optimization of concentration of TPTD with OCPcol will be needed before clinical applications are attempted. Early rat studies reported that osteosarcoma may occur if TPTD was given subcutaneously at 13.6 μg/kg/day for 2 years (total amount ∼10,000 μg/kg).^37^ However, the human studies indicated no increased risk of osteosarcoma, because the difference in findings in rat and human studies may be due to different patterns of use.^38^ In several preclinical studies with subcutaneous injection of TPTD, various amounts of TPTD were applied, from 10 μg/kg/day × 1 week (total amount 70 μg/kg)^39^ to 40 μg/kg/day × 9 weeks (total amount 2520 μg/kg).

The total amount of TPTD used in this study with OCPcol + TPTD was 4.61 ± 0.27 μg/kg, which was considerably lower than in other studies. Furthermore, this usage was similar to 3.97 ± 0.17 μg/kg, which was the previously reported level in the application of OCPcol + TPTD for a rodent critical-sized defect.^30^ Likewise, the usually administered doses of TPTD in patients with osteoporosis are ∼1 μg/kg/week (TERIBONE) or 0.2–0.5 μg/kg/day (Forteo^®^) and this provides a benchmark for the safe usage of TPTD. Hence, it must be verified that the safety and efficacy of OCPcol + TPTD are adequate for clinical applications, and the efficacy of a lower dose TPTD in OCPcol + TPTD should be investigated in the future.

## Conclusions

This study demonstrated that it was possible to achieve sufficient bone regeneration when single-dose local administration of TPTD with OCPcol was applied to intractable bone defects, such as segmental mandibular defects. Although several problems remain to be overcome before this treatment can be applied to clinical applications, it can be an option for the treatment of bone defects using OCPcol + TPTD, which was associated with physiological bone remodeling.

## References

[1] Dolderer, J.H., Geis, S., Mueller-Wille, R., *et al.* New reconstruction for bone integration of non-vascularized autogenous bone graft with better bony union and revascularisation. Arch Orthop Trauma Surg 137**,** 1451, 2017 28825132 10.1007/s00402-017-2775-y

[2] Kamei, Y., Nakayama, B., Toriyama, K., *et al.* Combined fibular osteocutaneous and omental flaps. Plast Reconstr Surg 119**,** 1499, 2007 17415244 10.1097/01.prs.0000256065.62382.89

[3] Nishida, J., and Shimamura, T. Methods of reconstruction for bone defect after tumor excision: a review of alternatives. Med Sci Monit 14**,** Ra107, 2008 18668007

[4] Warnke, P.H., Springer, I.N., Wiltfang, J., *et al.* Growth and transplantation of a custom vascularised bone graft in a man. Lancet 364**,** 766, 2004 15337402 10.1016/S0140-6736(04)16935-3

[5] Yamada, Y., Nakamura, S., Ito, K., *et al.* Injectable tissue-engineered bone using autogenous bone marrow-derived stromal cells for maxillary sinus augmentation: clinical application report from a 2-6-year follow-up. Tissue Eng Part A 14**,** 1699, 2008 18823276 10.1089/ten.tea.2007.0189

[6] Kokubo, T., Kim, H.M., and Kawashita, M. Novel bioactive materials with different mechanical properties. Biomaterials 24**,** 2161, 2003 12699652 10.1016/s0142-9612(03)00044-9

[7] Kikuchi, M., Itoh, S., Ichinose, S., Shinomiya, K., and Tanaka, J. Self-organization mechanism in a bone-like hydroxyapatite/collagen nanocomposite synthesized in vitro and its biological reaction in vivo. Biomaterials 22**,** 1705, 2001 11396873 10.1016/s0142-9612(00)00305-7

[8] Tsuchiya, A., Sotome, S., Asou, Y., *et al.* Effects of pore size and implant volume of porous hydroxyapatite/collagen (hap/col) on bone formation in a rabbit bone defect model. J Med Dent Sci 55**,** 91, 2008 19845154

[9] Sotome, S., Ae, K., Okawa, A., *et al.* Efficacy and safety of porous hydroxyapatite/type 1 collagen composite implantation for bone regeneration: a randomized controlled study. J Orthop Sci 21**,** 373, 2016 26961287 10.1016/j.jos.2016.01.007

[10] Garcia-Gareta, E., Coathup, M.J., and Blunn, G.W. Osteoinduction of bone grafting materials for bone repair and regeneration. Bone 81**,** 112, 2015 26163110 10.1016/j.bone.2015.07.007

[11] Kamakura, S., Sasano, Y., Shimizu, T., *et al.* Implanted octacalcium phosphate is more resorbable than beta-tricalcium phosphate and hydroxyapatite. J Biomed Mater Res 59**,** 29, 2002 11745534 10.1002/jbm.1213

[12] Anada, T., Kumagai, T., Honda, Y., *et al.* Dose-dependent osteogenic effect of octacalcium phosphate on mouse bone marrow stromal cells. Tissue Eng Part A 14**,** 965, 2008 19230123 10.1089/ten.tea.2007.0339

[13] Kamakura, S., Sasano, Y., Homma, H., Suzuki, O., Kagayama, M., and Motegi, K. Implantation of octacalcium phosphate nucleates isolated bone formation in rat skull defects. Oral Dis 7**,** 259, 2001 11575878

[14] Kamakura, S., Sasaki, K., Honda, Y., Anada, T., and Suzuki, O. Octacalcium phosphate combined with collagen orthotopically enhances bone regeneration. J Biomed Mater Res B Appl Biomater 79**,** 210, 2006 16615073 10.1002/jbm.b.30531

[15] Kouketsu, A., Matsui, K., Kawai, T., *et al.* Octacalcium phosphate collagen composite stimulates the expression and activity of osteogenic factors to promote bone regeneration. J Tissue Eng Regen Med 14**,** 99, 2020 31721475 10.1002/term.2969 PMC7027853

[16] Kamakura, S., Sasaki, K., Homma, T., *et al.* The primacy of octacalcium phosphate collagen composites in bone regeneration. J Biomed Mater Res A 83**,** 725, 2007 17559110 10.1002/jbm.a.31332

[17] Matsui, K., Matsui, A., Handa, T., *et al.* Bone regeneration by octacalcium phosphate collagen composites in a dog alveolar cleft model. Int J Oral Maxillofac Surg 39**,** 1218, 2010 20863660 10.1016/j.ijom.2010.07.015

[18] Kawai, T., Matsui, K., Iibuchi, S., *et al.* Reconstruction of critical-sized bone defect in dog skull by octacalcium phosphate combined with collagen. Clin Implant Dent Relat Res 13**,** 112, 2011 19438952 10.1111/j.1708-8208.2009.00192.x

[19] Kawai, T., Echigo, S., Matsui, K., *et al.* First clinical application of octacalcium phosphate collagen composite in human bone defect. Tissue Eng Part A 20**,** 1336, 2014 24294829 10.1089/ten.tea.2013.0508 PMC3993018

[20] Kawai, T., Tanuma, Y., Matsui, K., Suzuki, O., Takahashi, T., and Kamakura, S. Clinical safety and efficacy of implantation of octacalcium phosphate collagen composites in tooth extraction sockets and cyst holes. J Tissue Eng 7**,** 2041731416670770, 2016 27757220 10.1177/2041731416670770 PMC5051665

[21] Kawai, T., Suzuki, O., Matsui, K., Tanuma, Y., Takahashi, T., and Kamakura, S. Octacalcium phosphate collagen composite facilitates bone regeneration of large mandibular bone defect in humans. J Tissue Eng Regen Med 11**,** 1641, 2017 26612731 10.1002/term.2110

[22] Miura, K.I., Sumita, Y., Kajii, F., Tanaka, H., Kamakura, S., and Asahina, I. First clinical application of octacalcium phosphate collagen composite on bone regeneration in maxillary sinus floor augmentation: a prospective, single-arm, open-label clinical trial. J Biomed Mater Res B Appl Biomater 108**,** 243, 2020 30980703 10.1002/jbm.b.34384

[23] Kamakura, S. Development and clinical application of octacalcium phosphate/collagen composites. In: Suzuki, O., and Insley G., eds. Octacalcium Phosphate Biomaterials. 1st ed. Cambrige, MA, USA: Elsevier, 2020. pp. 289–308

[24] Kawai, T., Kamakura, S., Matsui, K., *et al.* Clinical study of octacalcium phosphate and collagen composite in oral and maxillofacial surgery. J Tissue Eng 11**,** 2041731419896449, 2020 32030119 10.1177/2041731419896449 PMC6978823

[25] Sibai, T., Morgan, E.F., and Einhorn, T.A. Anabolic agents and bone quality. Clin Orthop Relat Res 469**,** 2215, 2011 21132409 10.1007/s11999-010-1722-9 PMC3126945

[26] Niall, H.D., Sauer, R.T., Jacobs, J.W., *et al.* The amino-acid sequence of the amino-terminal 37 residues of human parathyroid hormone. Proc Natl Acad Sci U S A 71**,** 384, 1974 4521809 10.1073/pnas.71.2.384 PMC388010

[27] Morimoto, T., Kaito, T., Kashii, M., *et al.* Effect of intermittent administration of teriparatide (parathyroid hormone 1-34) on bone morphogenetic protein-induced bone formation in a rat model of spinal fusion. J Bone Joint Surg Am 96**,** e107, 2014 24990981 10.2106/JBJS.M.01097

[28] Tam, C.S., Heersche, J.N., Murray, T.M., and Parsons, J.A. Parathyroid hormone stimulates the bone apposition rate independently of its resorptive action: differential effects of intermittent and continuous administration. Endocrinology 110**,** 506, 1982 7056211 10.1210/endo-110-2-506

[29] Jilka, R.L., Weinstein, R.S., Bellido, T., Roberson, P., Parfitt, A.M., and Manolagas, S.C. Increased bone formation by prevention of osteoblast apoptosis with parathyroid hormone. J Clin Invest 104**,** 439, 1999 10449436 10.1172/JCI6610 PMC408524

[30] Kajii, F., Iwai, A., Tanaka, H., Matsui, K., Kawai, T., and Kamakura, S. Single-dose local administration of teriparatide with a octacalcium phosphate collagen composite enhances bone regeneration in a rodent critical-sized calvarial defect. J Biomed Mater Res B Appl Biomater 106**,** 1851, 2018 28922546 10.1002/jbm.b.33993 PMC6032915

[31] Iwai, A., Kajii, F., Tanaka, H., *et al.* Bone regeneration by freeze-dried composite of octacalcium phosphate collagen and teriparatide. Oral Dis 24**,** 1514, 2018 29943386 10.1111/odi.12923

[32] Ohba, S., Sumita, Y., Umebayashi, M., *et al.* Onlay bone augmentation on mouse calvarial bone using a hydroxyapatite/collagen composite material with total blood or platelet-rich plasma. Arch Oral Biol 61**,** 23, 2016 26492524 10.1016/j.archoralbio.2015.10.012

[33] Suzuki, O., Kamakura, S., Katagiri, T., *et al.* Bone formation enhanced by implanted octacalcium phosphate involving conversion into ca-deficient hydroxyapatite. Biomaterials 27**,** 2671, 2006 16413054 10.1016/j.biomaterials.2005.12.004

[34] Matsui, A., Matsui, K., Handa, T., *et al.* The regenerated bone quality by implantation of octacalcium phosphate collagen composites in a canine alveolar cleft model. Cleft Palate Craniofac J 51**,** 420, 2014 23369014 10.1597/12-096

[35] Miura, K., Matsui, K., Kawai, T., *et al.* Octacalcium phosphate collagen composites with titanium mesh facilitate alveolar augmentation in canine mandibular bone defects. Int J Oral Maxillofac Surg 41**,** 1161, 2012 22727604 10.1016/j.ijom.2012.05.020

[36] Beltran, M.J., Collinge, C.A., and Gardner, M.J. Stress modulation of fracture fixation implants. J Am Acad Orthop Surg 24**,** 711, 2016 27579811 10.5435/JAAOS-D-15-00175

[37] Watanabe, A., Yoneyama, S., Nakajima, M., *et al.* Osteosarcoma in sprague-dawley rats after long-term treatment with teriparatide (human parathyroid hormone (1-34)). J Toxicol Sci 37**,** 617, 2012 22688001 10.2131/jts.37.617

[38] Taylor, A.D., and Saag, K.G. Anabolics in the management of glucocorticoid-induced osteoporosis: an evidence-based review of long-term safety, efficacy and place in therapy. Core Evid 14**,** 41, 2019 31692480 10.2147/CE.S172820 PMC6711555

[39] Rowshan, H.H., Parham, M.A., Baur, D.A., *et al.* Effect of intermittent systemic administration of recombinant parathyroid hormone (1-34) on mandibular fracture healing in rats. J Oral Maxillofac Surg 68**,** 260, 2010 20116693 10.1016/j.joms.2009.09.045

